# Temperature assessment study of ex vivo holmium laser enucleation of the prostate model

**DOI:** 10.1007/s00345-022-04041-z

**Published:** 2022-05-25

**Authors:** Mehmet Yilmaz, Cäcilia Elisabeth Maria Heuring, Franz Friedrich Dressler, Rodrigo Suarez-Ibarrola, Christian Gratzke, Arkadiusz Miernik, Simon Hein

**Affiliations:** 1 Faculty of Medicine, Department of Urology, University of Freiburg—Medical Centre, Hugstetter Str. 55, 79106 Freiburg, Germany; 2 Faculty of Medicine, Institute of Pathology, University Medical Center Schleswig Holstein Lübeck Campus, Ratzeburger Allee, 23538 Lübeck, Germany

**Keywords:** Holmium laser, Prostate enucleation, Temperature, Thermal effect

## Abstract

**Purpose:**

There isscarce evidence to date on how temperature develops during holmium laser enucleation of the prostate (HoLEP). We aimed to determine the potential heat generation during HoLEP under ex vivo conditions.

**Methods:**

We developed two experimental setups. Firstly, we simulated HoLEP ex vivo using narrow-neck laboratory bottles mimicking enucleation cavities and a prostate resection trainer. Seven temperature probes were placed at different locations in the experimental setup, and the heat generation was measured separately during laser application. Secondly, we simulated high-frequency current-based coagulation of the vessels using a roller probe.

**Results:**

We observed that the larger the enucleated cavity, the higher the temperature rises, regardless of the irrigation flow rate. The highest temperature difference with an irrigation flow was approximately + 4.5 K for a cavity measuring 100ccm and a 300 ml/min irrigation flow rate. The higher flow rate generates faster removal of the generated heat, thus cooling down the artificial cavity. Furthermore, the temperature differences at different irrigation flow rates (except at 0 ml/min) were consistently below 5 K. Within the resection trainer, the temperature increase with and without irrigation flow was approximately 0.5 K and 3.0 K, respectively. The mean depth of necrosis (1084 ± 176 µm) achieved by the roller probe was significantly greater when using 144 W energy.

**Conclusion:**

Carefully adjusted irrigation and monitoring during HoLEP are crucial when evacuating the thermal energy generated during the procedure. We believe this study of ours provides evidence with the potential to facilitate clinical studies on patient safety.

**Supplementary Information:**

The online version contains supplementary material available at 10.1007/s00345-022-04041-z.

## Introduction

Holmium laser enucleation of the prostate (HoLEP) has become an increasingly popular minimally invasive prostate operation over the last 20 years [1]. HoLEP is an alternative totransurethral resection of the prostate (TURP) and open prostatectomy (OP) in its efficacy, safety, and in minimising complications, and it can be carried out regardless of the prostate’s size [2–5]. HoLEP has recently become the gold standard at various medical centres [6], mainly due to its superior surgical outcomes, lower bleeding risk, and fewer postoperative complications.

With excellent absorption properties in water resulting from a wavelength of 2140 nm, holmium:YAG (Ho:YAG) laser generates a very low penetration depth of approximately 0.4 mm. The energy emitted is transferred to hydrous prostate tissue [7]. Studies measuring the temperature development in various laser lithotripsy ex vivo models allow very few conclusions about how temperature develops during HoLEP [8–12], and until now, there has been no investigation of temperature development during HoLEP.

In this study, we therefore aimed to determine the potential heat generation during HoLEP under ex vivo conditions. We designed two experimental setups for this purpose. We first simulated HoLEP ex vivo using narrow-neck laboratory bottles as enucleation cavities, and measured the heat generation during Ho:YAG laser application. We then simulated high-frequency electric current-based coagulation of the vessels using a roller probe and assessed the depth of necrosis.

## Materials and methods

### *Experiment I*’s setup

The setup of *Experiment I* entailed ex vivo HoLEP surgery (Fig. 1—Online Resource 1). Narrow-neck laboratory bottles with different volumes (10, 20, 30, 50 and 100 cc) served as prostate enucleation cavities (see Fig. 1A, B—Online Resource 1). These were placed in a container filled with water using a rack that simulated the container´s temperature of the surrounding human pelvis. The pump (Reglo-Z Digital from Cole Parmer, Vernon Hills, Illinois, USA) generated a constant water flow of 100 ml/min in the container, which mimicked the convection effect of blood. The Ho:YAG laser Sphinx^®^ 100 W (LISA OHG, Katlenburg-Lindau, Germany), the 26 Fr continuous irrigation Shark series resectoscope (Richard Wolf GmbH, Knittlingen, Germany), and the 550 µm RigiFib laser fibre (LISA, Katlenburg-Lindau, Germany) corresponded to the standard HoLEP instruments utilised in the Department of Urology at the University Medical Centre Freiburg. Different irrigation flow rates (0, 300, 400 and 500 ml/min) were set using an irrigation pump (the Urology Pump (LUT GmbH, Denzlingen, Germany)). Since we were mainly concerned with the temperature difference, tap water at room temperature was used as the irrigation fluid. The laser was used throughout at 80 W power, single pulse energy of 4 J, and a pulse duration of 400 µs. Seven temperature probes (Pico Technology, Cambridgeshire, UK) were placed at different locations in the experimental setup (Figs. 1A, B): probe 1 in the post-enucleation cavity approximately 2 cm from the laser tip, probe 2 in the enucleation cavity approximately 5 cm from the laser tip, probe 3 on the proximal instrument shaft, probe 4 on the medial instrument shaft, probe 5 on the distal instrument shaft, probe 6 in the irrigation fluid before starting the experiment, and probe 7 in the irrigation fluid after the experiment. The temperature change during laser application was measured in each differently sized cavity at four different flow rates. The Thermocouple Data Logger USB TC-08 (Pico Technology, Cambridgeshire, UK) device was connected to a PC via a USB port. The temperature data measured were obtained using PicoLog 6 software. Each measurement with flush flow was taken at least three times. Each experiment without flushing flow was carried out once until a maximum temperature of approximately 60 °C was reached. The mean values of the respective measurements were recorded. Temperature differences (in *K*) are stated because the real values are irrelevant and enable a better overview. We relied on the largest measured difference in each case.

A different experimental setup was also investigated: here, the enucleation cavity was not in a water bath as before, rather, it was created in a realistic and commercially available prostate resection training model (Resection-Trainer TUR-P/BT LS10 3.0, Samed GmbH Dresden, Germany) (see Fig. 1C- Online Resource 1). This device is suitable for transurethral interventions simulating prostate procedures. For this purpose, probe 2 was placed in the simulated prostate parenchyma instead of in the enucleation cavity, approximately 5 cm away from the enucleation cavity. Temperature measurements were also recorded by an FLIR SC-640 (FLIR Systems, Portland, USA) thermal imaging camera during the experiment.

To conduct these experiments with a representative irrigation flow rate, the irrigation flow rates and other relevant parameters as laser application time and energy delivered had been initially determined during two HoLEP operations at the Department of Urology at the University Medical Centre Freiburg. The mean value for the irrigation rate equalled 420.5 ml/min. We assumed an average flushing flow rate of 400 ml/min. During this experiment, we ran additional tests at flush flow rates of 300 ml/min and 500 ml/min; these were intended to represent realistic deviations.

### *Experiment II*’s setup

In *Experiment II*, we employed a piece of bovine tissue-simulating prostate tissue. The bovine tissue was placed in Purisole on a neutral electrode and processed using the probe at various power settings 6.5 sprayCOAG^®^ (corresponding to approximately 78 W) and 10 sprayCOAG^®^ (corresponding to approximately 144 W) (see Fig. 1D- Online Resource 1). A monopolar current was used, and derivation via a neutral electrode under the metal plate. For the coagulation procedure, a coagulation electrode (Richard Wolf GmbH, Knittlingen, Germany) was used connected to a VIO^®^ 3 electrosurgical unit (Erbe Elektromedizin GmbH, Tübingen, Germany). This instrument is routinely used in the Department of Urology at the University Medical Centre Freiburg for haemostasis after laser enucleation of the prostate [6]. Following the coagulation procedure, the bovine tissue was evaluated histopathologically by an experienced pathologist. The corresponding standard deviation (SD) was determined.

The irrigation pump generates an irrigation flow within a 30–500 ml range and operates within a pressure range of 15–150 mmHg. Manual re-measurements revealed a reduced flow rate: an actual flow of 287 ml/min at 300 ml/min, actually 344 ml/min at 400 ml/min, and at 500 ml/min actually 436 ml/min. We took these genuine flow rates into account during the experiment. To simulate a constant flow in the water-filled container, the Reglo-Z Digital pump was used. The rinsing fluid was removed from the basin and then immediately reintroduced at a flow rate of 100 ml/min.

The same endoscopy devices as in *Experiment I* were used*.* No camera was connected in *Experiment II*, as the experiments were monitored macroscopically.

## Results

### The results of *Experiment I*

#### Influence of the enucleation cavity

To demonstrate the influence of the enucleation cavity´s size, we compared the largest (100 cc) to the smallest (10 cc) enucleation cavity (Table 1- Online Resource 2) (see Fig. 2A- Online Resource 3). The maximum temperature occurs after about 2 min in the 100 ccm enucleation cavity, and after about 30 s in the 10 ccm cavity. Assessing the largest enucleation cavity, we recorded higher temperature differences at all temperature probes except probes 4 and 7. A temperature difference of 3.7 K was recorded at probe 1 in the enucleation cavity with 100 cc volume. In comparison, at the same probe in the 10 ccm enucleation cavity, we recorded a difference of 3.5 K. This corresponds to a relative deviation of 5.7%.

### Influence of the irrigation flow rate

The corresponding temperature differences (in *K*) at each temperature probe are also listed in Table 1. The level of the irrigation flow rate causes a noticeable difference in the temperature development within the enucleation cavity and on the instrument shaft (see Fig. 2B- Online Resource 3).

Table 2 (Online Resource 2) shows the absolute temperature values without irrigation flow. The maximum temperature of approximately 61 °C is reached in the smallest enucleation cavity (10 ccm) after about 40 s, and in the enucleation cavity with 50 ccm volume after more than 180 s. As it plateaus at about 48 °C in the largest enucleation cavity (100 ccm), a temperature exceeding 60 °C is not reached.

### Different positions of the temperature probes

The curves in Fig. 3A (Online Resource 4) exemplify the temperature development at all six temperature probes for a 100-cc enucleation cavity at an irrigation flow rate of 400 ml/min. We found that the temperature gradually drops between the enucleation cavity and the distal end of the instrument’s shaft. Higher temperatures were recorded with the medial temperature probe than with the proximal temperature probe in the other enucleation cavities with different volumes. At the start of laser application, the liquid´s temperature rose in our experimental setup’s drain hose. Table 3 (Online Resource 2) lists the temperature differences measured at probe 7 in the different enucleation cavities, each with an irrigation flow of 344 ml/min. These values become smaller as the enucleation cavity size increases.

### Additional experimental setup: prostate resection training model

We took measurements at 10 ccm and an irrigation flow rate of 344 ml/min in each case (see Fig. 3B-Online Resource 4), or at 10 ccm and without irrigation flow (see Fig. 3C-Online Resource 4). With irrigation flow, we detected a temperature rise of approximately 0.5 K within the simulated prostate during 4 min of continuous laser application. Without irrigation flow, the temperature increase after 4 min was approximately 3.0 K. Table 4 (Online Resource 2) shows the temperature differences with probe 2, both with and without irrigation flow.

### Thermal imaging camera

Measurements from the thermal-imaging camera during the *Experiment I* confirm the temperature development at the model´s various positions. The temperature of the continuous irrigation laser resectoscope amounted to 21.7 °C.

### The results of *Experiment II*

The depth of necrosis was examined histopathologically. Table 5 (Online Resource 2) shows that for the mean depth of necrosis, the standard deviations are significantly greater when using 144 W than 78 W.

## Discussion

Although there have been studies investigating the thermal effects of thulium and holmium:YAG lasers, the number of published studies on heat generation during HoLEP and its associated risks is insufficient [13, 14]. In the present study, we investigated the temperature development during HoLEP ex vivo employing two different experimental models. We showed that temperature increases occur in the enucleation cavity and on the instrument’s shaft when the laser is used continuously. All measured temperature differences were below 4 K and thus not in a tissue-damaging range. The average maximum temperature difference across the instrument shaft was approximately + 2.5 K. There is published evidence that tissue damage begins to worsen from 42.5 °C temperature [15], and that the first thermal injury in the canine urethra occurs at 43 °C [16]. The temperature increase in our study cannot be considered clinically relevant, especially for the penile shaft and surrounding tissue, i.e. the urethra, at least theoretically.

We demonstrate that the larger the enucleation cavity, the higher is the temperature increase, regardless of the irrigation flow rate. Only in measurements without irrigation flow was the increase higher, with smaller enucleation cavity. The greatest temperature difference (approximately + 4.5 K) with irrigation flow was recorded with an enucleation cavity of 100 ccm at an irrigation flow rate of 300 ml/min. We can assume that under genuine surgical conditions, the absolute temperature rises to approximately 41.5 °C after < 3 min (in conjunction with an irrigation fluid temperature of about 37 °C (body temperature) in vivo. This value is below the presumed tissue damage limit according to the CEM43 (cumulative equivalent minutes at 43 °C) value and is not clinically relevant [17]. Therefore, HoLEP at an irrigation flow rate > 300 ml/min is feasible and safe in patients under surgical conditions.

The existing studies have mainly investigated ex vivo temperature generation during laser application, but with different experimental setups and aims. In an in vitro model, Hein et al. investigated the thermal effects of Tm:YAG laser treatment of the prostate [13]. They found a 15 K temperature increase in the urethra and up to 7 K parenchymal temperature increase using continuous laser application at 120 W and a 125 ml/min irrigation flow rate. In a more realistic experimental setup (30 s laser application followed by 30 s stop) performed in their study, they observed a rapid increase in urethral temperature during laser application with 80 W and even 50 ml/min irrigation, followed by an equally rapid decrease once the laser was stopped. In an ex vivo study by Kallidonis et al., the temperature development was measured in NaCl solution using the thulium:YAG laser. They found a larger rise in temperature especially at higher wattage, and that the temperature also increases significantly at lower flow rates [18].

In another ex vivo model, Aldoukhi et al. investigated temperature generation in liquid using the holmium laser at powers between 5 and 40 W. The highest temperature they measured was approximately 70 °C at 40 W and no irrigation flow. No temperature > 39 °C was measured at the highest irrigation flow rate [19]. The study by Maxwell et al. employed a computer-simulated model using the renal pelvis, calyxes and ureter. They found that injurious temperatures were at 5–40 W laser power without irrigation,  > 10 W with 5 mL/min irrigation, and 40 W with 15 mL/min irrigation. The maximum temperature without irrigation with 40 W was 70 °C on average. There were no injurious temperatures at a 40 mL/min irrigation rate, and no temperature > 37 °C was measured at the highest flushing flow rate [11]. Similar to these studies, we observed that the irrigation rate influenced how temperature develops in our HoLEP model. We showed that the higher flow rate enables faster removal of the generated heat, and thus cooling within the enucleation cavity. The temperature differences measured at different irrigation flow rates (except at 0 ml/min) stayed consistently below 5 K.

Relatively lower laser powers and higher irrigation flow rates appear to result in less heat generation during laser use. In their ex vivo study, Molina et al. investigated the development of temperature in a sheep ureter during laser lithotripsy using a human stone and Ho:YAG laser with a power of 10 W. They measured a significant increase in temperature at the level of the urothelium and on the outer ureteral wall, but this was significantly lower when using irrigation [12]. In their ex vivo model, Buttice et al. investigated temperature development in the kidney using the Holmium laser with and without irrigation fluid, at a power range of 5–20 W. Without rinsing fluid, the maximum temperature of 45 °C was reached in all experimental setups, regardless of the laser setting. No temperature increase above 1 K was recorded when using flushing fluidd [9]. Hein and Petzold investigated temperature development using a holmium laser in an ex vivo model of laser lithotripsy. They reported that a powerful setting combined with a low irrigation flow rate results in a rapid and clinically relevant temperature rise [10].

In *Experiment I*, we simulated prostate tissue using a realistic prostate resection trainer. Temperature probes within the resection model were used to record the temperature change there. Due to the long laser time required, we only conducted this experiment with a certain enucleation cavity size (10 ccm) with and without irrigation flow, and it was not repeated. In the measurement with irrigation flow, we observed a 0.5 K temperature increase after approximately 4 min. In the experiment without irrigation flow, the temperature increase was 3 K after 4 min. Both differences can be considered safe for patients. A systematic error may have been made using narrow-neck laboratory bottles, as they may have caused too much isolation of the liquid. Moreover, a continuous laser time of 4 min without irrigation flow is unrealistic in a genuine operating context, since the surgeon has to keep interrupting the laser’s activity to scrutinise the region in which he is operating, and failure of the irrigation fluid is noticed rapidly due to the disruptance in the field of view.

*In Experiment II,* we demonstrated that the necrosis at higher wattage is significantly deeper than at a lower wattage. This is attributable to more energy being delivered at a higher wattage, which therefore penetrates deeper into the tissue. However, as the prostate capsule consisting primarily of connective tissue—which is significantly thicker—we can assume an insignificant amount of deep damage being spread to relevant anatomical structures such as the vascular nerve bundles. Nevertheless, in vivo studies are needed to confirm these findings.

Our study has some limitations. Model measurements can only approximate actual operating conditions. The materials we used obviously deviate in their properties from actual organs. For example, narrow-neck laboratory bottles used as prostate enucleation cavities in *Experiment I* cannot fully represent actual conditions, so that the temperature regulation is altered due to a possibly thicker vessel wall. The irrigation flow rates also deviated from those previously established due to the inaccuracy of the irrigation pump used. However, accurate irrigation flow rates cannot be established in vivo either, as the adjustment is done manually and deviations are unavoidable. The flushing flow rates used in our model should therefore be considered primarily in terms of their general tendency and not as absolute values. Random errors were reduced by repetitions of the measurements, so that these can be ruled out. Nevertheless, systematic errors are possible. In *Experiment II*, the bovine tissue pieces we used can only approximately replicate periprostatic tissue. The lack of blood supply to the tissue may also have caused deviations. Repeating our experiments was infeasible due to the circumstances, but could have contributed to greater reproducibility.

## Conclusions

We show that the temperature development during laser application in an experimental ex vivo setup depends on the rate of irrigation flow. Furthermore, the size of the prostate enucleation cavity is relevant. To remove thermal energy, it is essential to carefully adjust and monitor irrigation during the HoLEP procedure. The irrigation flow rate can be controlled via an irrigation pump. Further innovations in this field such as a real-time monitoring system are therefore desirable and very clinically relevant. We believe that this study has the potential to facilitate clinical studies on patient safety.

## Supplementary Information

Below is the link to the electronic supplementary material.

Fig.1 **A** Overview of our experimental setup and positions of the temperature probes. a- Probe 1 in the post-enucleation cavity. b- Probe 2 in the enucleation cavity. c- Probe 3 on the proximal instrument shaft. d- Probe 4 on the medial instrument shaft. e- Probe 5 on the distal instrument shaft. f- Probe 7 in the irrigation fluid after the experiment. **B** Position of the temperature probes in the enucleation cavity and on the instrument shaft. a- Probe 1 in the enucleation cavity 2 cm to laser tip. b- Probe 2 in the enucleation cavity 5 cm to laser tip. c- Probe 3 on the proximal instrument shaft. **C** a- Probe 1. b- Probe 2. c- Probe 3. d- Probe 4. e- Resection trainer. **D** a- Bovine tissue. b- Coagulation electrode. c- a neutral electrode under the metal plat (PDF 319 KB)

Supplementary file2 (PDF 105 KB)

Fig.2 Experiment I (**A**) Temperature differences comparison of 100 ccm and 10 ccm enucleation cavities. **B** Influence of different flushing flow rates in a 100 ccm enucleation cavity (PDF 124 KB)

Fig.3 Temperature measurements: (**A**) Different positions of the temperature probes, example measurement at 100 ccm and 344 ml/min. **B** Resection trainer at 10 ccm and an irrigation flow rate of 344 ml/min. **C** Resection trainer at 10 ccm, initially no irrigation flow, after 2.5 min 344 ml/min (PDF 159 KB)

## Data Availability

The raw data is with the corresponding author and can be provided on request.

## Code availability

Not applicable for this section.
