# Supplementary figures and images for: Temperature assessment study of ex vivo holmium laser enucleation of the prostate model
Source: World J Urol. 2022 May 25;40(7):1867–72. doi: 10.1007/s00345-022-04041-z (PMC9236967; doi:10.1007/s00345-022-04041-z)

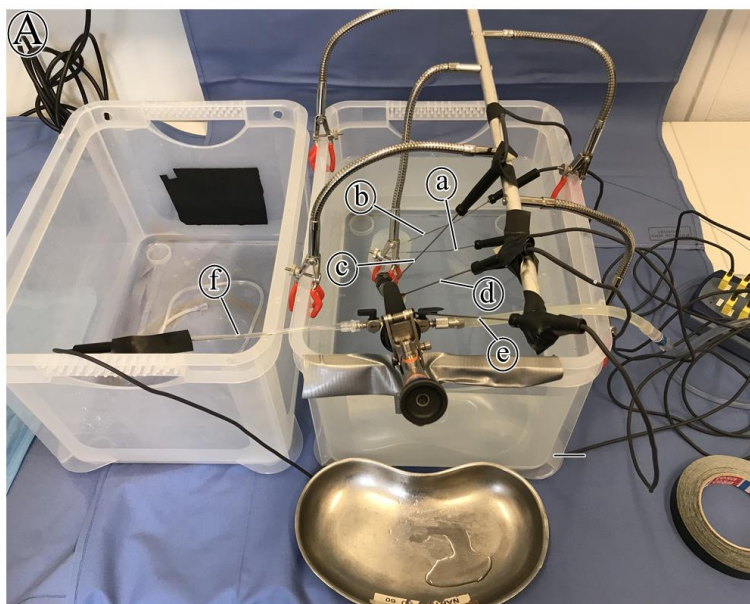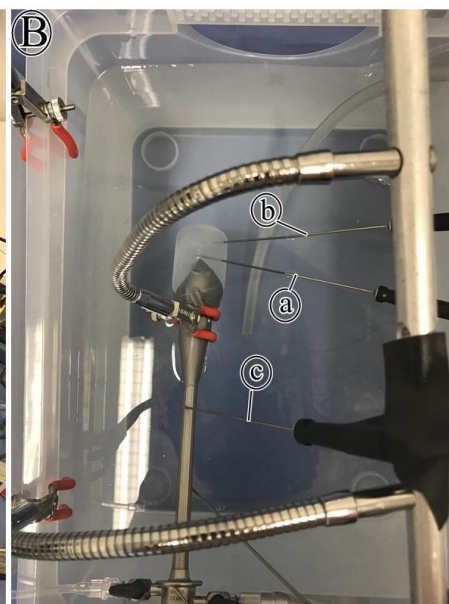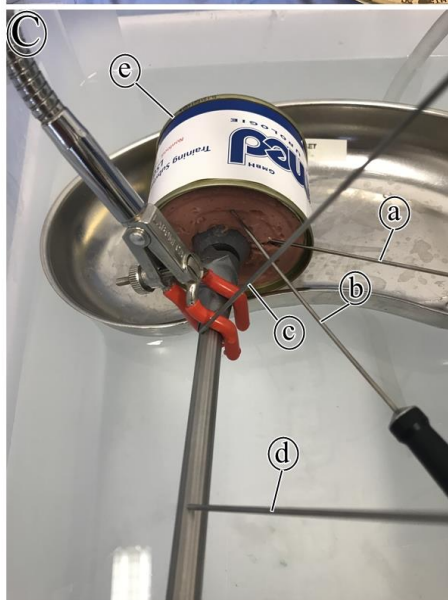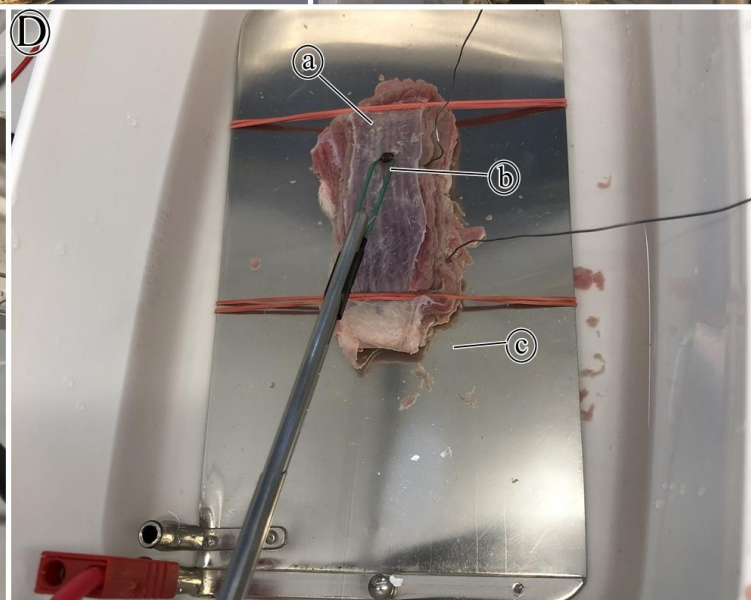

Supplement: Supplementary file 1 — Fig.1 A Overview of our experimental setup and positions of the temperature probes. a- Probe 1 in the post-enucleation cavity. b- Probe 2 in the enucleation cavity. c- Probe 3 on the proximal instrument shaft. d- Probe 4 on the medial instrument shaft. e- Probe 5 on the distal instrument shaft. f- Probe 7 in the irrigation fluid after the experiment. B Position of the temperature probes in the enucleation cavity and on the instrument shaft. a- Probe 1 in the enucleation cavity 2 cm to laser tip. b- Probe 2 in the enucleation cavity 5 cm to laser tip. c- Probe 3 on the proximal instrument shaft. C a- Probe 1. b- Probe 2. c- Probe 3. d- Probe 4. e- Resection trainer. D a- Bovine tissue. b- Coagulation electrode. c- a neutral electrode under the metal plat (PDF 319 KB) [file 345_2022_4041_MOESM1_ESM.pdf]

A

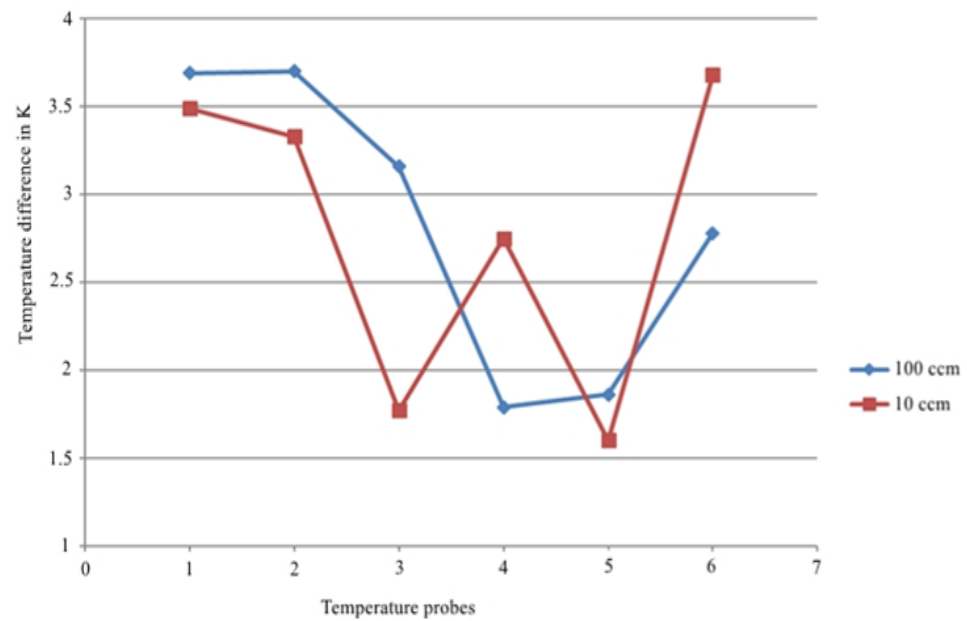

B

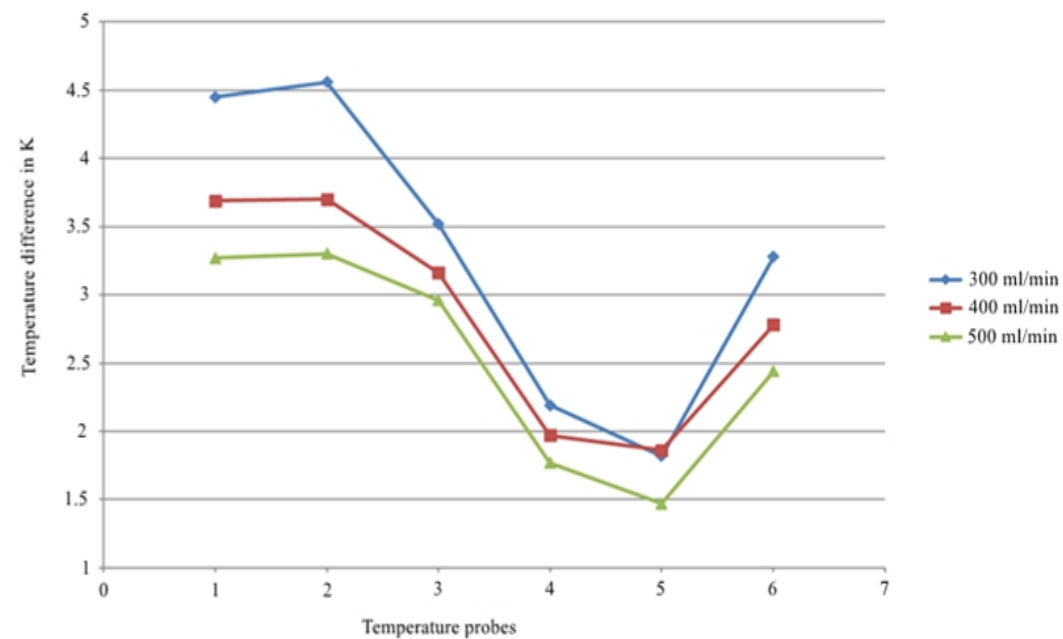

Supplement: Supplementary file 3 — Fig.2 Experiment I (A) Temperature differences comparison of 100 ccm and 10 ccm enucleation cavities. B Influence of different flushing flow rates in a 100 ccm enucleation cavity (PDF 124 KB) [file 345_2022_4041_MOESM3_ESM.pdf]
