# Supplementary material for: Temperature assessment study of ex vivo holmium laser enucleation of the prostate model
Source: World J Urol. 2022 May 25;40(7):1867–72. doi: 10.1007/s00345-022-04041-z (PMC9236967; doi:10.1007/s00345-022-04041-z)
Supplement: Supplementary file 2 — Supplementary file2 (PDF 105 KB) [file 345_2022_4041_MOESM2_ESM.pdf]

Table 1: Influence of the enucleation cavity and different irrigation flow rates in a 100 ccm enucleation cavity

Enucleation cavity	Probe 1	Probe 2	Probe 3	Probe 4	Probe 5	Probe 7
	Post-enucleation cavity	enucleation cavity	proximal instrument shaft	medial instrument shaft	distal instrument shaft	irrigation fluid after the experiment
100 ccm	3,7	3,7	3,2	1,8	1,9	2,8
10 ccm	3,5	3,3	1,8	2,8	1,6	3,7
Irrigation flow rate in 100 ccm						
287 ml/min	4,5	4,6	3,5	2,2	1,8	3,3
344 ml/min	3,7	3,7	3,2	2,0	1,9	2,8
436 ml/min	3,3	3,3	2,0	1,8	1,5	2,4

*Temperature difference in K

Table 2: Trials without irrigation flow

	After 10 s	After 20 s	After 30 s	After 60 s	After 120 s	After 180 s	Max- T
10 ccm	33 °C	47,0 °C	53,4 °C	-	-	-	61,9 °C
20 ccm	30,9 °C	36,5 °C	43,1 °C	55,8 °C	-	-	61,2 °C
30 ccm	26,9 °C	30,8 °C	34,9 °C	44,2 °C	57,7 °C	-	60,6 °C
50 ccm	25,6 °C	29,0 °C	31,4 °C	38,0 °C	49,2 °C	58,4 °C	62,0 °C
100 ccm	22,8 °C	25,2 °C	26,1 °C	29,8 °C	35,2 °C	38,9 °C	48,0 °C

T: Temperature

Table 3: Temperature differences Probe 7, outlet flushing

	Temperature difference (in K) –Probe 7 Flushing drain
10 ccm, 344 ml/min	3,68
20 ccm, 344 ml/min	3,32
30 ccm, 344 ml/min	3,39
50 ccm, 344 ml/min	3,16
100 ccm, 344 ml/min	2,78

Table 4: Prostate resection trainer, temperature differences (in K) of temperature probe 2, two irrigation flow rates in comparison for a enucleation cavity of 10 ccm

Prostate resection trainer	Temperature difference after					
	1 min	2 min	3 min	4 min	5 min	6 min
Probe 2, 344 ml/min	+/- 0 K	0,1 K	0,3 K	0,5 K	0,8 K	1,1 K
Probe 2, 0 ml/min	0,1 K	1,0 K	2,2 K	3,0 K	-	-

Table 5: Influence of power on necrosis depth in Experiment II

	6,5 sprayCOAG® in accordance with 78 W, n = 22	10 sprayCOAG® in accordance with 144 W, n = 8
Average depth of necrosis ± SD (µm)	718 ± 92	1084 ± 176

SD: standart deviation
