# Supplementary material for: Temperature assessment study of ex vivo holmium laser enucleation of the prostate model
Source: World J Urol. 2022 May 25;40(7):1867–72. doi: 10.1007/s00345-022-04041-z (PMC9236967; doi:10.1007/s00345-022-04041-z)
Supplement: Supplementary file 4 — Fig.3 Temperature measurements: (A) Different positions of the temperature probes, example measurement at 100 ccm and 344 ml/min. B Resection trainer at 10 ccm and an irrigation flow rate of 344 ml/min. C Resection trainer at 10 ccm, initially no irrigation flow, after 2.5 min 344 ml/min (PDF 159 KB) [file 345_2022_4041_MOESM4_ESM.pdf]

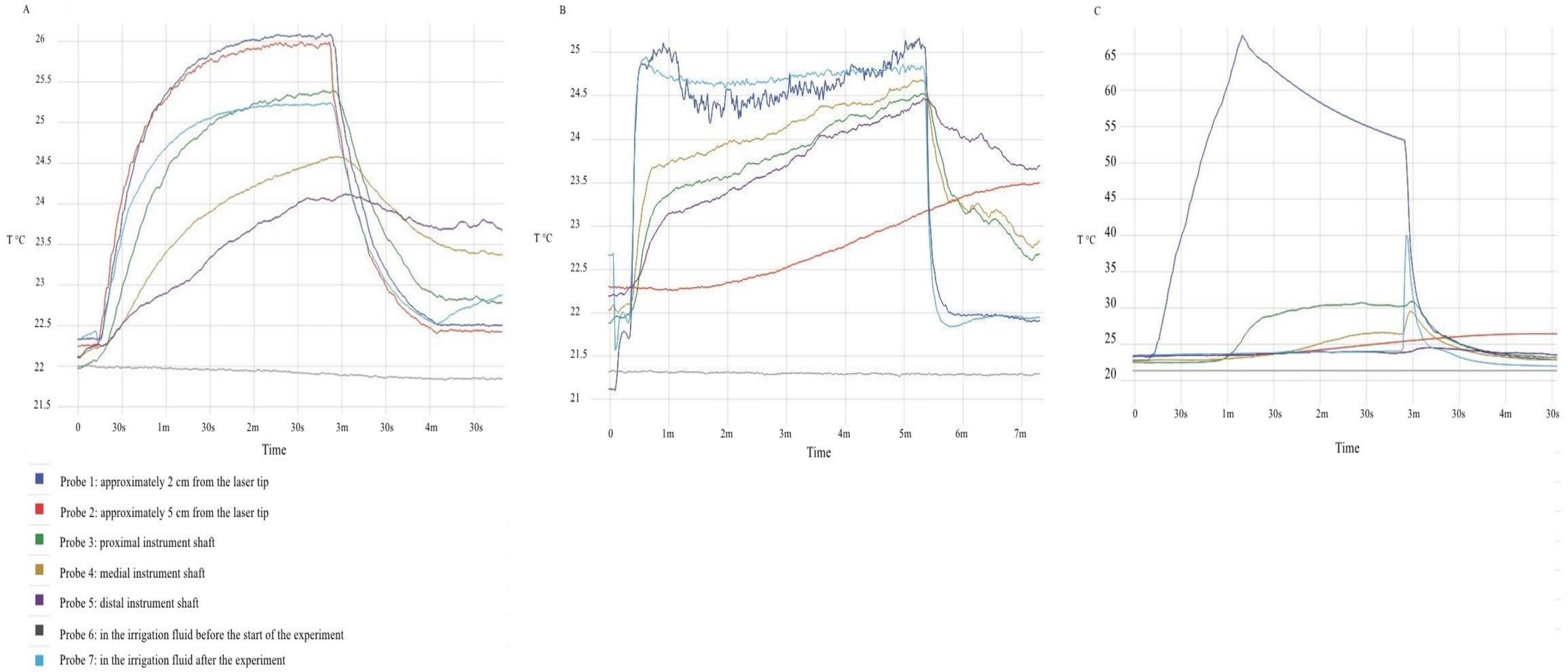
A
B
C
T °C
Time
Probe 1: approximately 2 cm from the laser tip
Probe 2: approximately 5 cm from the laser tip
Probe 3: proximal instrument shaft
Probe 4: medial instrument shaft
Probe 5: distal instrument shaft
Probe 6: in the irrigation fluid before the start of the experiment
Probe 7: in the irrigation fluid after the experiment
